# Metabolic Rewiring in Tea Plants in Response to Gray Blight Disease Unveiled by Multi-Omics Analysis

**DOI:** 10.3390/metabo13111122

**Published:** 2023-11-01

**Authors:** Shiqin Zheng, Zhenghua Du, Xiaxia Wang, Chao Zheng, Zonghua Wang, Xiaomin Yu

**Affiliations:** 1 Tea Research Institute, Fujian Academy of Agricultural Sciences, Fuzhou 350013, China; azhyeah@163.com; 2 Center for Plant Metabolomics, Haixia Institute of Science and Technology, Fujian Agriculture and Forestry University, Fuzhou 350002, China; zhenghuadu@fafu.edu.cn (Z.D.); wangxiaxia530@fafu.edu.cn (X.W.); zhengchaotea@fafu.edu.cn (C.Z.); 3 State Key Laboratory of Ecological Pest Control for Fujian and Taiwan Crops, College of Plant Protection, Fujian Agriculture and Forestry University, Fuzhou 350002, China; 4 Fuzhou Institute of Oceanography, Minjiang University, Fuzhou 350108, China

**Keywords:** tea plant, gray blight disease, flavonoid, lignin, defense-related metabolites, metabolomics, transcriptomics

## Abstract

Gray blight disease, which is caused by *Pestalotiopsis*-like species, poses significant challenges to global tea production. However, the comprehensive metabolic responses of tea plants during gray blight infection remain understudied. Here, we employed a multi-omics strategy to characterize the temporal transcriptomic and metabolomic changes in tea plants during infection by *Pseudopestalotiopsis theae*, the causal agent of gray blight. Untargeted metabolomic profiling with ultra-performance liquid chromatography–quadrupole time-of-flight mass spectrometry (UPLC-QTOFMS) revealed extensive metabolic rewiring over the course of infection, particularly within 24 h post-inoculation. A total of 64 differentially accumulated metabolites were identified, including elevated levels of antimicrobial compounds such as caffeine and (−)-epigallocatechin 3-gallate, as well as oxidative catechin polymers like theaflavins, theasinensins and theacitrins. Conversely, the synthesis of (+)-catechin, (−)-epicatechin, oligomeric proanthocyanidins and flavonol glycosides decreased. Integrated omics analyses uncovered up-regulation of phenylpropanoid, flavonoid, lignin biosynthesis and down-regulation of photosynthesis in response to the pathogen stress. This study provides novel insights into the defense strategies of tea plants against gray blight disease, offering potential targets for disease control and crop improvement.

## 1. Introduction

The tea plant (*Camellia sinensis*) is an important cash crop cultivated primarily for its leaves, which are used as raw material for tea production. China is the world’s largest tea producer and exporter, with an annual production of tea leaves exceeding 13 million tons in 2021, supporting the livelihoods of millions in the tea industry [1]. However, tea production faces persistent challenges from fungal diseases [2]. *Pestalotiopsis-like* species, known as the causal agents of gray blight disease in tea plants, are a highly destructive group of phytopathogens [3].

*Pestalotiopsis*-like species, which are classified into *Pseudopestalotiopsis*, *Neopestalotiopsis* and *Pestalotiopsis*, infect both tender shoots and mature leaves [4]. The initial symptoms of gray blight appear as small brown concentric spots on wounded leaves, which gradually expand into large necrotic lesions with black and brown colors. In severe cases, these lesions spread throughout the leaf, leading to defoliation [5]. Disease severity is exacerbated under warm and humid conditions, negatively impacting tea yield and quality [2]. Various *Pestalotiopsis*-like species have caused significant yield losses in major tea-producing countries like China, India and Japan [6,7,8]. Current disease management relies heavily on synthetic fungicides such as methyl benzimidazole carbamates and dithiocarbamates. However, these approaches come with high costs, environmental concerns and an increased risk of fungal resistance development [2,9,10]. Therefore, developing effective control measures requires a profound understanding of the defense mechanisms employed by the tea plant against the causal agent of gray blight disease.

Plants have evolved a sophisticated innate immune system to fend off pathogens through multilayered defense responses [11]. Physical structures such as thorns, trichomes, rigid cell walls and waxy cuticles form the first line of defense by preventing pathogen entry. In addition to these physical barriers, plants can recognize invading pathogens and initiate complex signaling cascades that trigger the targeted production of pathogenesis-related proteins, enzymes and specialized metabolites [12]. Among these metabolites are antimicrobial compounds, including constitutively expressed phytoanticipins and induced phytoalexins synthesized de novo in response to infection. These metabolites can directly inhibit microbial growth or elicit additional immune responses, serving to contain pathogens within the infected plant tissues [13]. Tea plants (*Camellia sinensis*), in particular, reply on the production of specialized metabolites (e.g., phenolics, alkaloids and terpenes) as a key defense strategy against biotic and abiotic stresses [14]. Phenolic compounds, such as (−)-epigallocatechin (EGC), (−)-epicatechin (EC) and their gallate esters, play a crucial role in leaf resistance. Among these phenolics, (−)-epigallocatechin 3-gallate (EGCG), the most abundant catechin in fresh tea leaves, is particularly noteworthy. EGCG has been shown to possess potent antibacterial, antifungal and antiviral properties [15,16]. Through a multifaceted mechanism of action, EGCG effectively combats fungi via disruption of cell structures, inhibition of key enzymes and synergistic potentiation of antifungal drugs, enabling robust activity against fungal invaders [16]. When infected by pathogens such as *Colletotrichum fructicola* or *Pseudopestalotiopsis* isolates, tea plants up-regulate EGCG or other catechin levels in correlation with enhanced resistance [17,18]. Nonetheless, the full scope of the specialized metabolic adaptation of tea plants during infection remains poorly understood.

In our previous study, we identified a novel pathogenic strain, *Pseudopestalotiopsis theae* CYF27, as the cause of gray blight disease in tea plants [19]. To further explore the molecular responses of tea plants to *P. theae* infection, we conducted an integrated multi-omics analysis. Through untargeted metabolomics, we characterized the temporal dynamics of the tea plant metabolome during *P. theae* infection. Additionally, through RNA sequencing, we uncovered transcriptional reprogramming events that underlie the immune response. This integrated omics approach provides new perspectives on the intricate host–pathogen interactions over time and may help inform the development of sustainable disease control strategies.

## 2. Materials and Methods

### 2.1. Chemical and Reagents

MS-grade acetonitrile and methanol were purchased from Thermo Fisher Scientific, Inc. (Pittsburgh, PA, USA), and formic acid was obtained from Honeywell Fluka (Seelze, Germany). Ultra-pure water was prepared with a Milli-Q purification system (Millipore, Bedford, MA, USA). Reference compounds were obtained from Sigma-Aldrich (St. Louis, MO, USA), ChemFaces (Wuhan, China), Yuanye Biotechnology Inc. (Shanghai, China) and BioBioPha Co., Ltd. (Kunming, China).

### 2.2. Plant Materials, Pathogen Inoculation and Sampling

One-year-old tea seedlings (*C. sinensis* cv. “Tieguanyin”) were obtained from a tea plantation in Anxi, Fujian, China (118°13′ E, 25°08′ N). The seedlings were cultivated hydroponically using Hoagland’s nutrient solution (pH 5.6) in a greenhouse at Fujian Agriculture and Forestry University (Fuzhou, China) under the following conditions: 25 ± 3 °C, 16 h photoperiod and 65 ± 5% relative humidity. To ensure the optimal growth of tea seedlings, the nutrient solution was aerated using an oxygen pump and replaced every seven days. After growing for over 30 days, seedlings that displayed uniform growth and were free from any signs of disease or insect infestation were selected as test materials.

The pathogenic strain *P. theae* CYF27, which has been previously reported [19], was used for inoculation in this study. CYF27 was first incubated at 25 °C on potato dextrose agar (PDA) medium for 20 days. PDA discs (6 mm) containing mycelia and conidia were excised to serve as the inocula. Prior to inoculation, tea leaves were rinsed with sterile water and air-dried to remove any residues from the surface. Each tea seedling received inoculation at three fully expanded leaves with two inoculation sites per leaf. Each site was gently scratched three times (~5 mm in length) with a sterile needle. A 5 mm disc of PDA medium bearing mycelia and conidia was placed onto the upper surface of the wounded tea leaf. The inoculated leaves were covered with cotton dampened in sterile water and wrapped in plastic. The seedlings were then transferred to an inoculation chamber (26 ± 3 °C, 16 h photoperiod, 90 ± 5% relative humidity) for 24 h of dark incubation. At 48 h post-inoculation, the coverings were removed to allow the seedlings to continue growing until harvest. The control samples were wounded tea leaves inoculated with blank PDA discs of the same size (hereafter referred as “CK”). For metabolome and transcriptome analyses, the entire fungal-inoculated leaves (hereafter referred as “PT”), along with the corresponding whole leaves from the CK samples, were harvested at 0, 1, 3 and 6 days post-inoculation (dpi) in three independent biological replicates. Each replicate consisted of three individual seedlings. Samples were snap-frozen in liquid nitrogen and subsequently stored at −80 °C for further analyses.

### 2.3. Metabolite Extraction and UPLC-QTOFMS Analysis

To prepare samples for metabolite analysis, the freeze-dried samples were ground to fine powders with pre-chilled mortars and pestles. Approximately 30 mg of leaf powder was weighted and extracted with 1.0 mL of 70% aqueous methanol. The resulting mixture was subjected to sonication for 20 min, followed by centrifugation at 12,000× *g* for 10 min. The supernatant was then filtered using a 0.22 μm PVDF filter (Millipore).

The metabolomics data were acquired on a Waters Acquity UPLC system (Milford, MA, USA) coupled with a Waters SYNAPT G2-S*i* HDMS QTOF mass spectrometer (Manchester, UK). The instrument was operated in electrospray ionization (ESI) mode and controlled with MassLynx 4.2 software (Waters). UPLC separation was carried out on a Waters Acquity UPLC HSS T3 column (1.8 μm, 2.1 × 100 mm). The mobile phase consisted of water containing 0.1% formic acid (Solvent A) and acetonitrile containing 0.1% formic acid (Solvent B). Gradient elution was performed as per the following program: 0–2 min (1–7% B), 2–13 min (7–40% B), 13–14 min (40–99% B), 14–18 min (held at 99% B) and 5 min of re-equilibration time before the next injection. The CK and PT samples were injected randomly. The ESI parameters were as follows: source temperature, 100 °C; desolvation temperature, 350 °C; cone gas flow, 50 L/h; desolvation gas flow, 800 L/h; capillary voltage, 1.28 kV; sampling cone voltage, 40 V. Data were acquired in both negative and positive ionization modes, operating in a full-scan mode over a mass range of 50–1200 *m*/*z*. The MS^e^ data were collected in the continuum mode with a collision energy ramp ranging from 10 to 50 eV. Online calibration of MS data was achieved by continually infusing leucine enkephalin (Waters, Milford, MA, USA) at a rate of 2 ng/min.

### 2.4. Metabolite Annotation and Multivariate Analysis

Progenesis QI software (v2.4, Nonlinear Dynamics) was used to process the raw data acquired in the positive and negative ionization modes separately. We detailed the process of data processing and metabolite identification in a prior study [20]. Briefly, raw data were imported into Progenesis QI for peak alignment, picking and normalization (with normalization to all compounds) using the default settings. To achieve the fusion of precursor ions, different adduct ion forms were grouped. These included [M−H]^−^, [2M−H]^−^ and [M+FA−H]^−^ in ESI^−^ and [M+H]^+^, [2M+H]^+^ and [M+Na]^+^ in ESI^+^. The generated matrix involving the information of retention time, *m*/*z* and normalized peak abundance from each mode was exported and combined for subsequent multivariate statistical analyses in Simca-P 14.1 software (Umetrics, Umea, Sweden). After *Pareto* scaling, principal component analysis (PCA) and orthogonal partial least-squares discriminant analysis (OPLS-DA) were applied to identify metabolites with significant differences between the CK and PT groups at each time point, based on a fold change (FC) threshold > 2, variable importance in projection (VIP) value > 1 and *p*-value < 0.05. The differential metabolites were annotated by referencing an in-house MS database as well as public databases such as Metlin, MassBank and HMDB [20,21,22,23].

### 2.5. Transcriptome Analysis

To explore the temporal changes in transcriptional response during *P. theae* infection, we performed RNA-seq analysis on pathogen-inoculated leaf samples harvested at 0, 1, 3 and 6 dpi (designated as PT0, PT1, PT3 and PT6, respectively). Briefly, total RNA was extracted using the RNAprep Pure Plant Kit (Tiangen, Beijing, China) according to the manufacturer’s instructions. Sequencing libraries were prepared using the NEBNext Ultra RNA Library Prep Kit for Illumina (NEB, Ipswich, MA, USA). The resulting library was then paired-end-sequenced (150 bp reads) on an Illumina HiSeq2500 platform (Novogene Biotech, Beijing, China) following standard protocols. The raw sequence data were deposited in the Genome Sequence Archive (GSA) database under the accession no. CRA011958.

After quality trimming, the clean reads were mapped to the reference transcriptome (*Camellia sinensis* cv. “Suchazao”) from the TPIA database (http://tpia.teaplants.cn, accessed on 16 June 2022) using the Salmon program (v0.9.0) [24] after the exclusion of reads that contained *P. theae* sequences. Gene expression was quantified as transcripts per million (TPM). DESeq2 was employed to identify differentially expressed genes (DEGs) with a false discovery rate (FDR) < 0.05 and |log_2_fold change| > 1. Gene ontology (GO) enrichment analysis were performed using the clusterProfiler package [25]. Kyoto Encyclopedia of Genes and Genomes (KEGG) enrichment analysis was performed using the KOBAS software [26].

### 2.6. Quantitative Real-Time PCR (qRT-PCR)

To validate the expression levels of DEGs, qRT-PCR was performed using SYBR Premix Ex Taq^TM^ II (Takara, Dalian, China) on a CFX96TM real-time PCR system (Bio-Rad, Hercules, CA, USA) as previously described [27]. Primers for 12 randomly chosen DEGs were designed using the Primer Premier software (v5.00) (Table S1). The relative expression of genes was determined using the 2^−∆∆Ct^ method [28] with the *CsGAPDH* gene (KA295375.1) as an endogenous control. Each biological sample was analyzed in triplicate.

### 2.7. Statistical Analysis

Data were analyzed using GraphPad Prism 8, TBtools (v.1.131) [29] and Microsoft Excel 2010. Results were reported as the mean ± standard deviation (SD) obtained from at least three replicates. The statistical discrepancy between treatments was evaluated using the nonparametric Mann–Whitney *U* test or one-way ANOVA with a post hoc Fisher’s least significant difference test as appropriate. In all cases, differences were considered significant at *p* < 0.05.

## 3. Results and Discussion

### 3.1. Morphological Analysis of Gray Blight Symptom Development in Tea Leaves

**Figure 1:**
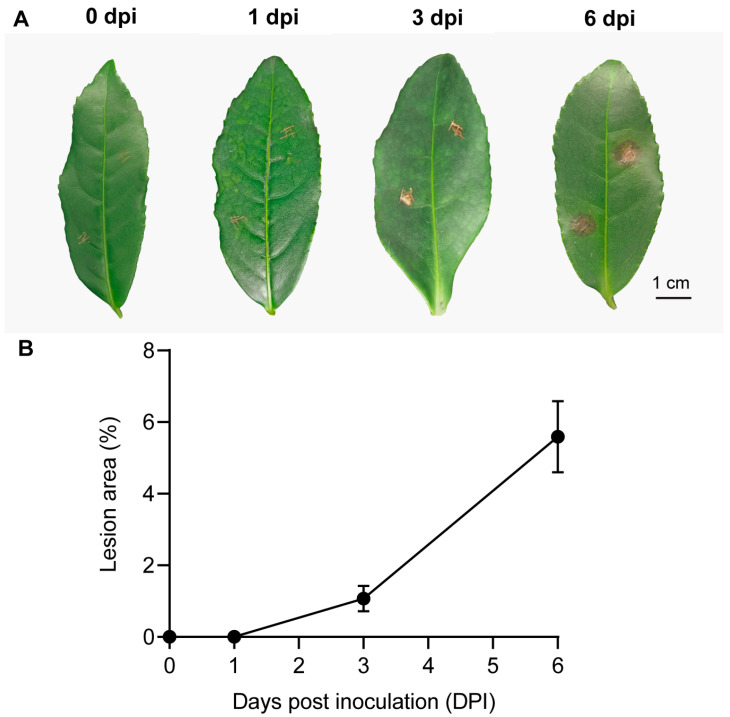
Development of gray blight disease on tea leaves over time. Intact leaves were inoculated with mycelia and conidia mixture of *P. theae*. (**A**) Representative symptoms on tea leaves using intact plant inoculation. Scale bar = 1 cm. (**B**) Statistics of disease development on tea leaves assessed at 0, 1, 3 and 6 dpi following fungal inoculation. The combined leaf disease area (cm^2^) from two inoculations per leaf was calculated as a percentage of the total leaf area using ImageJ software.

The onset and progression of disease symptoms on tea leaves inoculated with *P. theae* were monitored over the infection process (Figure 1A). At 0 and 1 dpi, no symptoms were observed on the wounded leaves. At 3 dpi, the symptoms began to manifest, appearing as small brown lesions specifically at the sites where the leaves had been pierced. These lesions gradually enlarged as the infection developed. By 6 dpi, the individual lesions merged together, forming prominent necrotic brown spots across the injured areas. Quantitative analysis showed that the lesion size increased by 4.5-fold from 3 to 6 dpi (Figure 1B). Interestingly, a similar study conducted on the “Suchazao” tea cultivar using a different *Pseudopestalotiopsis* isolate reported a similar lesion onset at 4 dpi, but the subsequent progression was much slower. In fact, it took 13 dpi for the lesions in “Suchazao” to coalesce into large brown spots resembling those observed at 6 dpi in our study [18]. This disparity implies that the rate at which symptoms spread may vary depending on the specific combination of the tea cultivar and the pathogenic *Pseudopestalotiopsis* isolate under investigation.

### 3.2. Metabolic Analysis of Tea Plant Response to P. theae Infection

**Figure 2:**
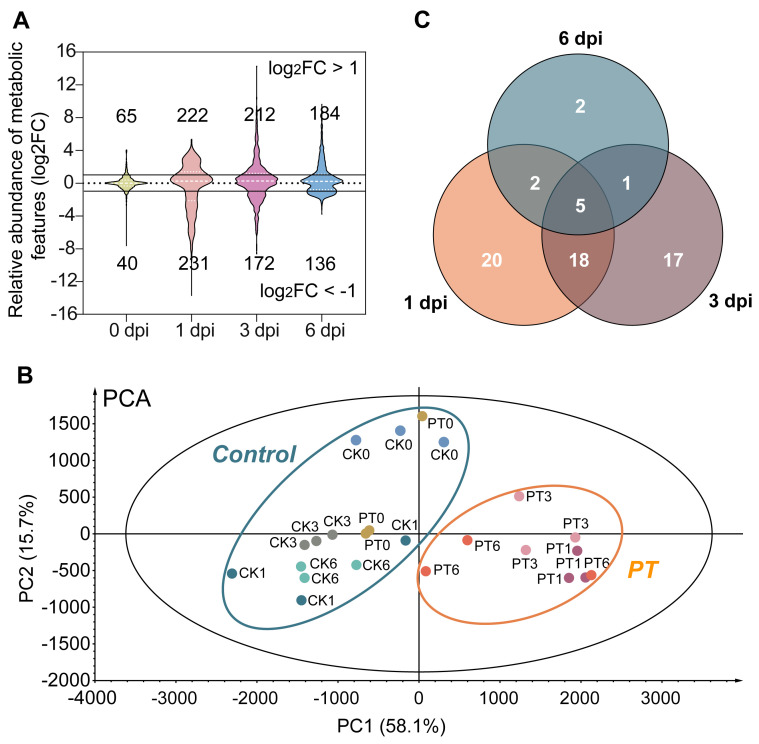
Time course analysis of tea metabolome reconfiguration in response to gray blight infection. (**A**) Overview of altered metabolic features. The violin plot shows the fold change values for the fungal-inoculated group relative to the mock-inoculated group at each time point. (**B**) Principal component analysis of tea leaves inoculated with *P. theae* or the mock control. (**C**) Venn diagram of differential metabolites between fungal-inoculated and mock-inoculated groups at each time point. PT, tea leaves inoculated with *P. theae*. CK, tea leaves inoculated with the mock control.

The changes in catechin contents in tea leaves afflicted by gray blight disease have been explored previously [18]. However, the impact of *P. theae* infection on other defense-related metabolites (e.g., phytoanticipins and phytoalexins) in tea plants remains largely unknown. To gain comprehensive insights into how tea metabolism is altered during pathogenesis, we conducted non-targeted metabolomics profiling of tea leaves subjected to *P. theae* inoculation or mock inoculation at 0, 1, 3 and 6 dpi. The representative total ion chromatograms of tea leaf metabolic fingerprints at 1 dpi in the negative mode are presented in Figure S1. After excluding fungal metabolites, we detected a total of 5362 metabolic features in the negative ionization mode and 3048 in the positive mode across all samples. Signals occurring at low abundance (maximum abundance < 1000) were removed, retaining 677 and 295 features in the negative and positive modes, respectively. Chemometrics analyses were then performed on the merged data from both modes. Our analysis unveiled dynamic metabolic shifts following fungal inoculation, as indicated by the log_2_-fold differences in the relative metabolite abundance between the infected groups and the control groups (Figure 2A). As expected, the metabolite profiles of tea samples at 0 dpi were similar between the CK and PT groups. However, at 1 dpi, the infected group displayed the most intense metabolic response among all time points examined, with sharp increases in both up-regulated and down-regulated metabolic features. Studies have shown that the early plant–pathogen interactions typically take place within 24 h post-inoculation, during which pathogens can fully invade and colonize the host tissues [30]. Hence, the direct contact between the tea plant and *P. theae* likely triggered the activation of the plant’s defense system at 1 dpi. When comparing the pathogen-inoculated group to the mock-inoculated group, there was an increased abundance of 222, 212 and 184 metabolic features and a decreased abundance of 231, 172 and 136 features at 1, 3 and 6 dpi, respectively (Figure 2A). From 1 to 6 dpi, the number of up-regulated features in the PT group compared to CK only slightly declined, whereas the number of down-regulated features decreased by 41%. This sustained up-regulation and lessening down-regulation of metabolic features in response to fungal infection suggest complex and dynamic interactions between the tea plant and the pathogen.

PCA analysis showed that, except for samples harvested at 0 dpi, the pathogen-inoculated samples were clearly distinguished from the mock-inoculated samples, with the first two principal components (PCs) explaining 73.8% of the total variance (Figure 2B). This again highlights the significant impact of *P. theae* infection on metabolite rewiring in tea plants. Within the PT group, one biological replicate harvested at 6 dpi deviated from the other two replicates, which made clustering by inoculation duration less distinct in the PCA plot.

To further elucidate the differences between the fungal-inoculated and mock-inoculated samples, we employed OPLS-DA analysis, a robust supervised multivariate method known for its ability to identify statistically significant variables [31]. The OPLS-DA score plots visually depict the separation between the sample groups based on their metabolite profiles at each time point (Figure S2). This analysis enabled us to observe distinct cluster patterns and confirm the significant metabolic alterations associated with the fungal infection. A total of 64 metabolites displayed statistically significant differences (*p* < 0.05, VIP > 1 and |FC| > 2), as shown in Table 1. These differentially accumulated metabolites (DAMs) were classified into 10 categories: polymerized catechin derivatives (23), flavonol glycosides (13), flavanols (8), flavone and glycosides (2), flavanone glycosides (1), xanthine alkaloids (1), amino acids (1), hydrolysable tannins (1), phenolic acids and derivatives (1) and unknown compounds (13). Flavonoid compounds made up the vast majority (72%) of the identified DAMs, pointing to their key roles in controlling gray blight infection.

A Venn diagram was constructed to visualize the overlap and differences in annotated DAMs between the treatment and control groups (Figure 2C). The number of DAMs showed a notable decrease as the infection progressed, with 45 identified at 1 dpi, 41 at 3 dpi and only 10 at 6 dpi. This indicates that the tea plant’s metabolic response was most pronounced immediately after pathogen invasion but tapered over time. This decline could signify a transition from acute defense to a more balanced state. Alternatively, the pathogen’s manipulation or suppression of certain metabolic pathways may contribute to the decrease. The metabolic alterations coincided with the phenotypic observation of progressively enlarged necrotic spots as the inoculation duration increased (Figure 1). Among the DAMs shared across all time points (Figure 2C and Table 1), four metabolites—caffeine, theaflavin 3,3′-digallate, EGCG and theacitrin C—showed a persistent induction in response to the *P. theae* challenge compared to CK. As a naturally occurring alkaloid prevalent in plants, caffeine functions as a broad-spectrum deterrent against herbivores and pathogens through its antimicrobial properties [32]. Notably, previous studies have demonstrated the ability of caffeine to inhibit the growth of the tea pathogens *P. theae* and *C. fructicola* even at low concentrations [17,33]. This underscores the probable essential role of caffeine in the innate defense response of tea plants to fungal infection. Similarly, reports from various plant–fungus interaction systems have documented a sustained increase in different flavonoid compounds as an integral component of innate immunity [34]. The consistent accumulation of these specialized metabolites in infected tea leaves throughout the infection process may suggest a strategic modulation of metabolism that imparts long-lasting resistance against pathogen challenges. Further evaluation is warranted to explore the potential of these metabolites as candidate markers of resistance and to elucidate how tea plants coordinate chemical defense.

### 3.3. Metabolic Reprogramming of Flavonoid Biosynthesis in Tea Plants against P. theae Infection

**Figure 3:**
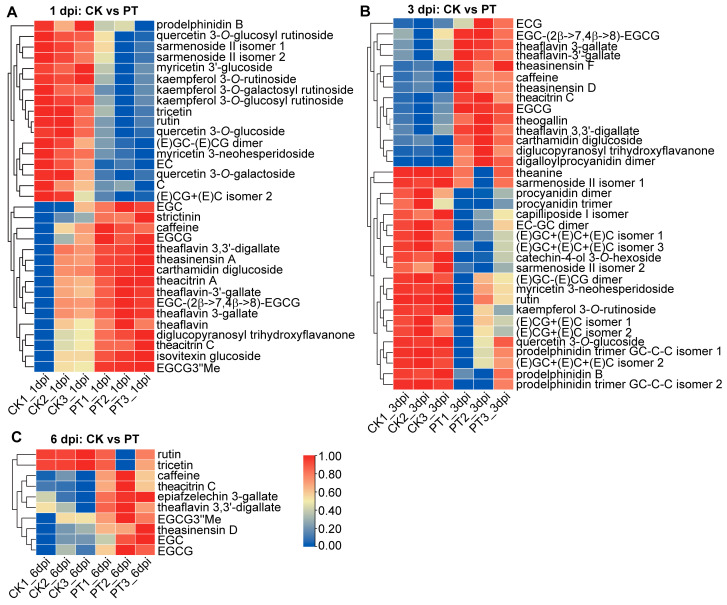
Heatmaps of annotated metabolites showing significant differences between fungal-inoculated and mock-inoculated tea leaves at 1 dpi (**A**), 3 dpi (**B**) and 6 dpi (**C**). Red and blue colors signify increased and decreased levels of metabolites. PT, tea leaves inoculated with *P. theae*. CK, tea leaves inoculated with the mock control.

The heatmap visualization of the annotated DAMs reveals fascinating patterns of flavonoid metabolism in tea plants upon pathogen attack (Figure 3A–C). During early infection (1 and 3 dpi), the levels of EGCG, (−)-epigallocatechin (EGC), (−)-epicatechin gallate (ECG) and (−)-epigallocatechin 3-*O*-(3-*O*-methyl)gallate (EGCG3″Me) notably increased, while the EC and C levels decreased. Tea polyphenols, with EGCG, EGC, ECG and EC being the dominant compounds in tea leaves, demonstrate effective antifungal properties by inhibiting the growth and spore germination of various phytopathogens [16]. Additionally, studies conducted in Kenya and India have indicated an inverse correlation between the content of tea polyphenols and susceptibility to the pathogen *P. theae* [35]. These studies suggest the potential of tea polyphenols as antifungal agents for disease control, although the contribution of each polyphenol component to the antifungal activity is yet to be established. Of note, our results differ from those reported by Wang et al., who observed increased EC and GC contents in gray blight-inflicted tea leaves at 4 dpi, with minimal changes in EGCG and ECG [18]. The reasons for the disparities remain unclear but could relate to differences in *P. theae* isolates and tea cultivars used in the two studies. Therefore, further studies are needed to comprehend the influence of these factors on catechin biosynthesis during fungal infection and to clarify the antifungal effects of individual catechins against *P. theae*.

In addition to alterations in catechin metabolism, the concentrations of various proanthocyanidin oligomers, including prodelphinidin B, procyanidin dimers, EC-GC dimer, (E)GC-(E)CG dimer and (E)GC-(E)C-(E)C trimer, decreased in tea leaves. This decline could be attributed to the depletion of their precursors, EC and C. There was also a marked reduction in the biosynthesis of flavonol glycosides, such as several derivatives of quercetin, kaempferol and myricetin, which are typically abundant in tea leaves. On the contrary, the concentrations of several oxidative polymerized products of catechins, namely theaflavins (theaflavin, theaflavin 3-gallate, theaflavin 3′-gallate and theaflavin 3, 3′-digallate), theasinensins (theasinensin A, D and F) and theacitrins (theacitrin A and C), increased alongside the elevated levels of their precursors, EGCG and EGC (Figure 3). There are two potential explanations for this accumulation. Firstly, these oxidative polymers might possess additional antifungal or antioxidant properties that directly contribute to the stress response against fungal infection. Alternatively, fungal infection could trigger oxidative stress in tea leaves through the enhanced activities of enzymes like peroxidase and polyphenol oxidase. Such oxidative stress may result in increased catechin polymerization. Although theaflavins, theasinensins and theacitrins are typically found in higher quantities during the fermentation of oolong and black teas, it is worth noting that a study detected increased levels of theaflavins in fresh tea leaves infested by tea green leafhoppers [36,37,38,39]. Nonetheless, the induction of oxidative catechin polymers as a response to biotic stresses has received little exploration. Understanding whether these polymers function directly as defensive compounds or are formed indirectly due to oxidative stress would enhance our comprehension of the metabolic defenses employed by tea plants.

**Figure 4:**
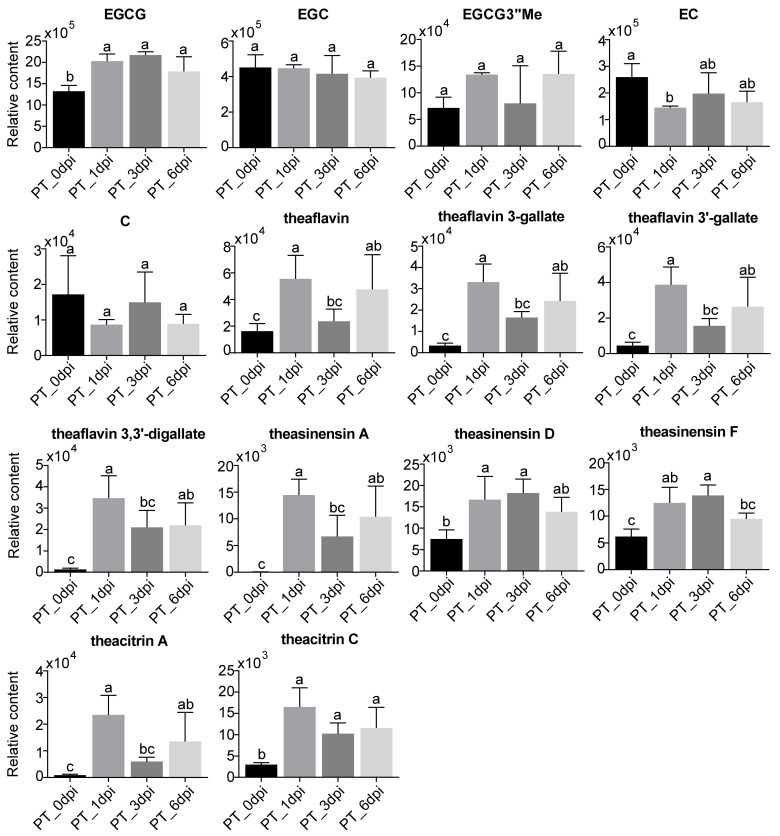
Quantitative analysis of catechins and polymerized catechin derivatives extracted from metabolomics data. The *y*-axis denotes peak area values obtained through normalization to the “normalize to all compounds function” in Progenesis QI. The relative abundance of each metabolite is presented as mean ± SD (*n* = 3). Different letters denote significant differences in metabolite contents at different time points, as determined by Fisher’s least significant difference test at *p* < 0.05.

To analyze the variation trends of catechin monomers and polymers during the course of infection, we plotted the relative contents of these compounds in fungal-infected tea samples (Figure 4). EGCG consistently showed a significant increase at 1 dpi (*p* < 0.05) and maintained higher levels until 6 dpi. The level of EC was significantly lower at 1 dpi compared to 0 dpi (*p* < 0.05). C levels also tended downward, albeit the change was not statistically significant. As for oxidized catechin polymers, the aforementioned theaflavins, theasinensins and theacitrins all showed a marked surge at 1 dpi and primarily remained elevated at subsequent time points despite some fluctuations.

Collectively, the results show that tea plants strategically reconfigure their flavonoid profiles in response to infection by prioritizing the production of potent antimicrobial flavanols (e.g., EGCG) while reallocating resources away from potentially less bioactive compounds (e.g., EC, C and flavonol glycosides). This orchestrated adjustment likely strengthens the defense against fungal pathogens. Our work offers unique perspectives into the intricate flavonoid-mediated responses of tea plants to control infection.

### 3.4. Transcriptomic Analysis of Tea Plant Response to P. theae Infection

**Figure 5:**
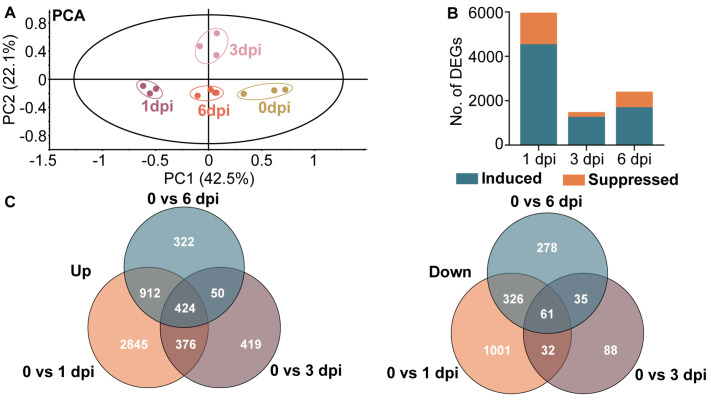
Time course analysis of tea transcriptome reprogramming in response to gray blight infection. (**A**) Principal component analysis of fungal-inoculated tea leaves at 0, 1, 3 and 6 dpi. (**B**) Number of differentially expressed genes (DEGs) at each time point. Teal and orange columns indicate up- and down-regulated genes, respectively. (**C**) Venn diagrams illustrating the number of DEGs up- or down-regulated due to fungal treatment over the time course.

To further understand the global transcriptome responses modulated by *P. theae* infection, RNA sequencing was performed on *P. theae*-inoculated tea leaves at 0, 1, 3 and 6 dpi. Detailed information on RNA sequencing is provided in Table S2. Overall, 330,641,626 clean reads were generated from 12 samples, and these reads were mapped to the reference genome of “Suchazao”, with alignment rates ranging between 86.00% and 89.61%. PCA analysis of the transcriptome data demonstrated clear clustering of biological replicates within each time point, providing evidence of *P. theae*-induced changes in gene expression (Figure 5A). The first two PCs collectively accounted for 64.6% of the total variance. Samples at 1 dpi were positioned at the far left of the score plot, whereas samples at 0 dpi were located at the far right along the first PC. This is concordant with the metabolomics data showing that the most drastic variation was induced at 1 dpi. A total of 29,037 transcripts were detected in all samples (Table S3). After filtering by FDR < 0.05 and absolute log_2_ (fold change) > 1, we identified 5967 DEGs (4547 up and 1420 down), 1485 DEGs (1269 up and 216 down) and 2408 DEGs (1708 up and 700 down) in PT_0 dpi vs. PT_1 dpi, PT_0 dpi vs. PT_3 dpi and PT_0 dpi vs. PT_6 dpi comparisons, respectively, suggesting that *P. theae* infection caused substantial transcriptional reprogramming in the tea plants (Figure 5B). In all comparisons, a higher number of genes were up-regulated compared to those that were down-regulated, which is consistent with the changes at the metabolomic level. In addition, many DEGs were unique to each time point, with 2845, 419 and 322 genes uniquely up-regulated and 1001, 88 and 278 genes uniquely down-regulated at 1, 3 and 6 dpi, respectively (Figure 5C). The reliability of the RNA-seq data was validated through qRT-PCR analysis, which confirmed the expression patterns of 12 randomly selected DEGs in the phenylpropanoid, flavonoid, shikimate and lignin pathways (Figure S3).

**Figure 6:**
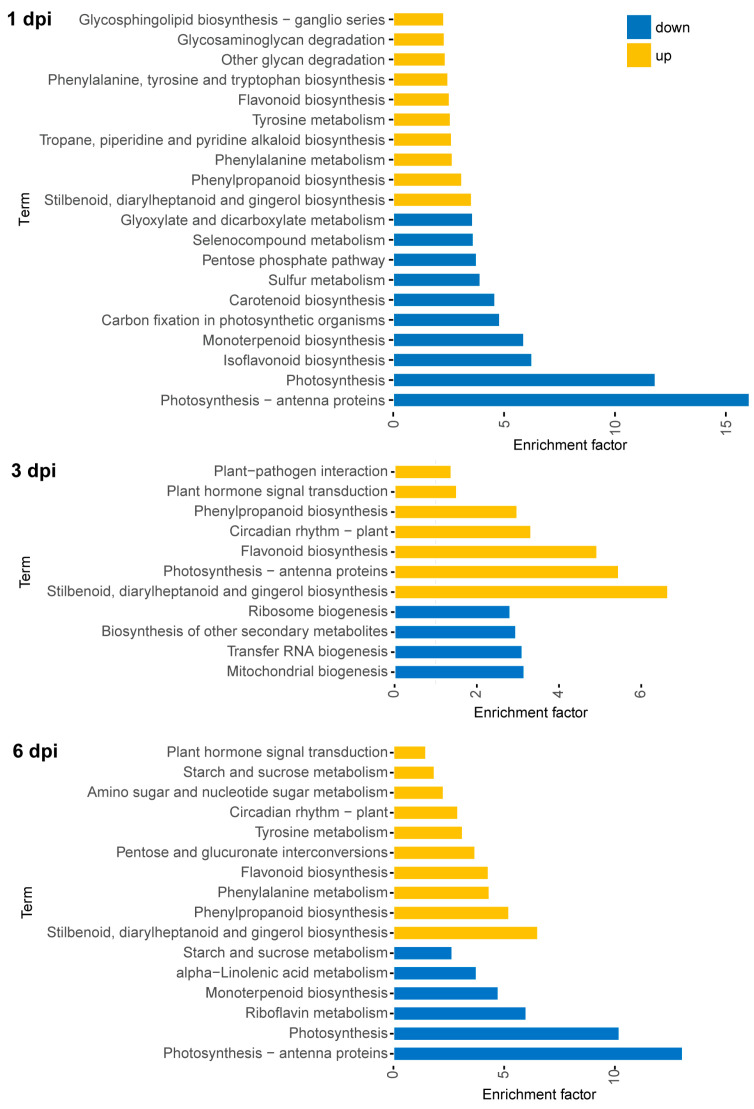
KEGG pathway enrichment analysis of DEGs following fungal treatment. The up-regulated pathways are in yellow, while the down-regulated pathways are in blue.

GO classification of DEGs was performed to categorize the unigenes into biological processes, molecular functions and cellular components (Figure S4). Within the biological process category, the DEGs were found to be abundant in GO terms such as “cellular process”, “single organism process”, “metabolic process” and “response to stimulus”. The molecular function category mainly comprised DEGs associated with “binding” and “catalytic activity”. In the cellular component category, highly represented groups included “cell”, “cell part”, “organelle” and “membrane”. We conducted KEGG pathway enrichment analysis on DEGs to investigate the primary metabolic pathways involved in tea plant response. The top 10 enriched up-regulated and down-regulated pathways at 1, 3 and 6 dpi are listed in Figure 6. Key enriched pathways included phenylpropanoid biosynthesis, flavonoid biosynthesis, photosynthesis, amino acid metabolism, starch and sucrose metabolism, plant–pathogen interaction and plant hormone signal transduction. Three pathways—“phenylpropanoid biosynthesis”, “flavonoid biosynthesis” and “photosynthesis-antenna proteins”—were commonly enriched across all time points. Previous studies have demonstrated that pathogen-infected plant leaves often experience a decrease in photosynthetic rates due to disturbances in the photosynthetic machinery [40]. In line with this, our studies also found that *P. theae* infection, particularly at 1 dpi, disrupted pathways related to photosynthesis, such as “photosynthesis-antenna proteins”, “carbon fixation in photosynthetic organisms” and “porphyrin and chlorophyll metabolism” (Table S4). The disruption of photosynthesis may have broader implications for plant defense, as the chloroplast plays a pivotal role in fortifying plant immunity through the synthesis of a diverse array of molecules, hormones and proteins that possess antimicrobial properties [41].

### 3.5. Transcriptomic Rewiring of Phenylpropanoid and Flavonoid Metabolism in Tea Plants in Response to P. theae Infection

KEGG pathways for phenylpropanoid and flavonoid biosynthesis were significantly enriched following infection (Figure 6). This correlates well with the elevated flavonoid levels from the metabolomics data, indicating the importance of these pathways in tea plant response to gray blight disease. Similar enrichment of phenylpropanoid and flavonoid biosynthesis was also observed in a prior study involving the interaction between the tea cultivar “Suchazao” and a pathogenic *Pseudopestalotiopsis* isolate [18]. Therefore, we closely examined the transcriptional profiles of genes involved in phenylpropanoid and flavonoid metabolism in tea leaves.

**Figure 7:**
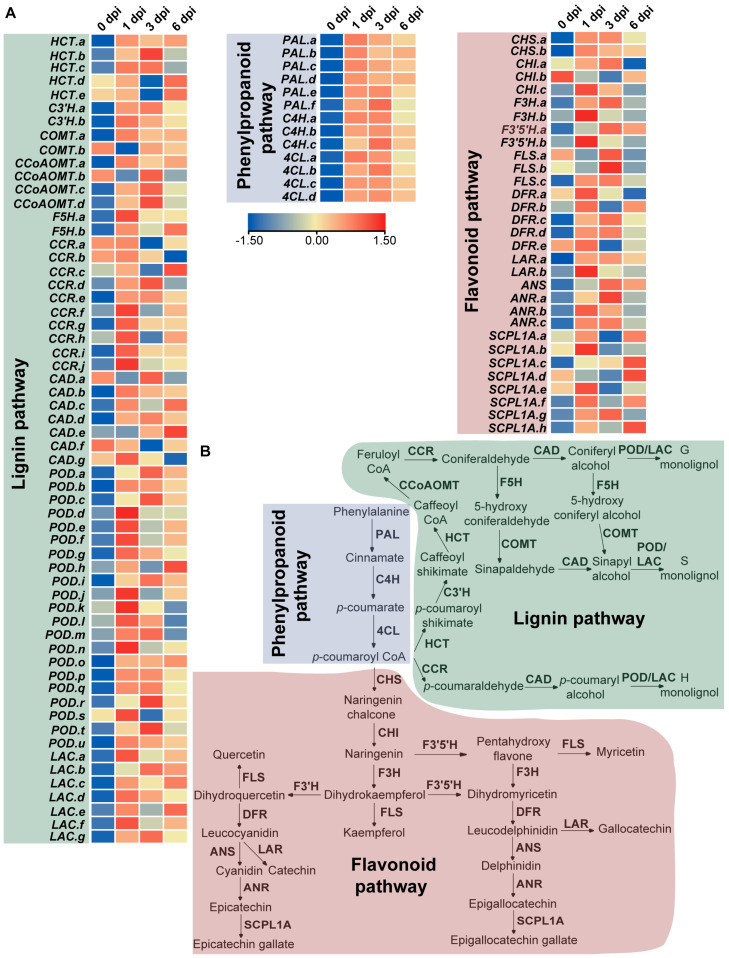
Expression profiles of genes involved in phenylpropanoid, lignin and flavonoid pathways in tea leaves after fungal treatment. (**A**) Heatmap comparison of the TPM values of related genes in three pathways based on RNA-seq data. (**B**) Simplified diagram of phenylpropanoid, lignin and flavonoid pathways. PAL, phenylalanine ammonia lyase; C4H, cinnamate 4-hydroxylase; 4CL, 4-coumarate-CoA ligase; CHS, chalcone synthase; CHI, chalcone isomerase; F3H, flavanone 3-hydroxylase; F3′H, flavanone 3′-hydroxylase; F3′5′H, flavanone 3′,5′-hydroxylase; FLS, flavonol synthase; DFR, dihydroflavonol 4-reductase; LAR, leucoanthocyanidin reductase; ANS, anthocyanidin synthase; ANR, anthocyanidin reductase; SCPL1A, type 1A serine carboxypeptidase-like acyltransferases; CCR, cinnamoyl-CoA reductase; HCT, hydroxycinnamoyl-CoA:shikimate/quinate hydroxycinnamoyl transferase; C3′H, *p*-coumaroyl quinate/shikimate 3′-hydroxylase; CCoAOMT, caffeoyl-CoA *O*-methyltransferase; CAD, cinnamyl alcohol dehydrogenase; COMT, caffeic acid O-methyltransferase; F5H, ferulate 5-hydroxylase; PER, peroxidase; LAC, laccase.

The role of phenylpropanoids as inducible antimicrobial compounds and signaling molecules in plant–pathogen interactions is well established [42]. Similarly, we observed pronounced increases in the transcription levels of genes encoding phenylalanine ammonia-lyase (PAL), cinnamate 4-hydroxylase (C4H) and 4-coumarate-CoA ligase (4CL) within the general phenylpropanoid pathway (Figure 7A). PAL, which catalyzes the initial committed step critical for regulating phenylpropanoid metabolism, showed up-regulation of all six transcripts at 1 dpi, with sustained high expression at late infection stages. C4H and 4CL function sequentially downstream to produce *p*-coumaroyl CoA, an essential precursor for the biosynthesis of various phenolic compounds, such as flavonoids and lignin monomers [43]. They showed similar expression patterns to PAL (Figure 7A,B).

With only some exceptions, genes in the flavonoid pathway were predominantly up-regulated in response to the pathogen at 1 dpi (Figure 7A). Metabolite profiling demonstrated differential accumulation of flavanols and flavonol glycosides. However, differential expression of genes responsible for directing metabolic flux towards specific branches of flavanols or flavonols was not evident. This disconnect between metabolite and transcriptional changes hints at the possibility of additional post-transcriptional controls in regulating flavonoid biosynthesis in tea plants challenged by *P. theae*.

Also being generated via the phenylpropanoid pathway, lignin comprises a highly branched polymer of monolignols that functions as a fundamental building block of plant cell walls. During the early defense response to pathogen infection (1 and 3 dpi), most lignin biosynthetic genes in tea leaves were up-regulated, though there were some instances of down-regulation for certain transcripts (Figure 7A). This is consistent with the well-documented role of lignin in enhancing plant tolerance to various biotic and abiotic stresses across plant species [44]. Aside from serving as a barricade for pests and pathogens, mounting evidence shows that cell wall lignin dynamically adjusts its concentration and composition in response to environmental stimuli, providing protection in a wide range of contexts [44,45,46]. The resilience of lignin against microbial degradation suggests that increased lignification may fortify the infected cells and restrict pathogen spread [45]. It should be noted that variations in lignin accumulation under fungal infection were not investigated in our study, and future qualitative and quantitative analyses of lignin dynamics will provide additional insights. Intriguingly, peroxidase and laccase transcripts universally showed up-regulation at 1 dpi (Figure 7A). These enzymes are actively involved in the polymerization of monolignols into lignin polymers [47]. Isolation and biochemical analysis of individual isoenzymes in the future experiments will disentangle their roles in lignin formation and pathogen protection.

## 4. Conclusions

Through integrated “omics” analyses, this study uncovered the dynamic global transcriptomic and metabolomic changes in tea plants challenged with *P. theae*. By employing untargeted metabolomics coupled with multivariate testing, we identified 64 differentially accumulated metabolites between infected and control groups across multiple time points, highlighting the significant impact of this pathogen on tea plant metabolism. Rapid and substantial metabolic rewiring was observed at 1 dpi, being characterized by enhanced synthesis of caffeine, EGCG and oxidative catechin polymers, as well as a reduction in EC, proanthocyanidins and flavonol glycosides. Combined analysis of metabolomic and transcriptomic data implicates the involvement of phenylpropanoid, flavonoid and lignin pathways in tea plant resistance. Notably, we reveal for the first time the differential modulation of different branches of the flavonoid biosynthetic pathway in the tea plant in response to fungal challenge. These findings shed light on the complex and dynamic interactions between the tea plant and the causal agent of gray blight. Furthermore, our study enables the identification of key defensive genes, metabolites and pathways with potential for disease resistance breeding. Given that resistance can vary among tea cultivars, future comparative studies exploring varieties with varying degrees of susceptibility will provide deeper insights into the resistance mechanisms and aid in the development of more effective strategies for disease management and crop improvement.

## Figures and Tables

**Table 1:** Identification or tentative identification of significantly differential metabolites in response to *P. theae* infection.

No.	Metabolite Assignment	RT (min)	Metabolite Class	Adducts	Formula	Theoretical *m*/*z*	Measured *m*/*z*	PPM Error	MS/MS Fragments	Time Point
1	theanine *	1.40	amino acids	[M−H]^−^	C_7_H_14_N_2_O_3_	173.0926	173.0931	2.89	155.0826, 128.0945	3 dpi
2	theogallin *	2.92	phenolic acids and derivatives	[M−H]^−^	C_14_H_16_O_10_	343.0665	343.0672	2.04	191.0568, 169.0143, 125.0244	3 dpi
3	prodelphinidin B	4.14	polymerized catechin derivatives	[M−H]^−^	C_30_H_26_O_14_	609.1244	609.1250	0.99	441.0826, 423.0716, 305.0665, 125.0242	1, 3 dpi
4	EGC-(2->7,4->8)-EGCG	4.16	polymerized catechin derivatives	[M−H]^−^	C_37_H_28_O_18_	759.1197	759.1191	−0.79	605.0929	1, 3 dpi
5	catechin-4-ol 3-*O*-hexoside	4.53	flavanols	[M−H]^−^	C_21_H_24_O_12_	467.1190	467.1188	−0.43	305.0659, 125.0246	3 dpi
6	prodelphinidin trimer GC-C-C isomer 1	4.74	polymerized catechin derivatives	[M−H]^−^	C_45_H_38_O_20_	897.1878	897.1879	0.11	771.1569	3 dpi
7	EC-GC dimer	4.81	polymerized catechin derivatives	[M+H]^+^	C_30_H_26_O_13_	595.1452	595.1440	−2.02	279.0922	3 dpi
8	theacitrin A	4.86	polymerized catechin derivatives	[M−H]^−^	C_37_H_28_O_18_	759.1197	759.1194	−0.40	741.1062, 571.0869	1 dpi
9	prodelphinidin trimer GC-C-C isomer 2	4.86	polymerized catechin derivatives	[M−H]^−^	C_45_H_38_O_20_	897.1878	897.1875	−0.33	771.158	3 dpi
10	epigallocatechin *	4.91	flavanols	[M−H]^−^	C_15_H_14_O_7_	305.0661	305.0666	1.64	179.0352, 125.0251	1, 6 dpi
11	unknown	4.93	unknown	[M−H]^−^	C_45_H_40_O_20_	899.2035	899.2035	0.00	ND	3 dpi
12	(E)GC+(E)C+(E)C isomer 1	5.14	polymerized catechin derivatives	[M−H]^−^	C_45_H_38_O_19_	881.1929	881.1926	−0.34	423.0722, 305.0671, 287.0561, 125.0248	3 dpi
13	catechin *	5.33	flavanols	[M−H]^−^	C_15_H_14_O_6_	289.0712	289.0718	2.08	179.0347, 137.0241, 123.0451	1 dpi
14	strictinin	5.34	hydrolysable tannins	[M−2H]^2−^	C_27_H_22_O_18_	633.0728	633.0730	0.32	316.0328, 300.9989, 275.0194	1 dpi
15	(E)GC+(E)C+(E)C isomer 2	5.56	polymerized catechin derivatives	[M−H]^−^	C_45_H_38_O_19_	881.1929	881.1927	−0.23	305.0649, 287.0550	3 dpi
16	caffeine *	5.56	alkaloids	[M+H]^+^	C_8_H_10_N_4_O_2_	195.0882	195.0893	5.64	138.0671, 110.0720	0, 1, 3, 6 dpi
17	procyanidin dimer	5.68	polymerized catechin derivatives	[M−H]^−^	C_30_H_26_O_12_	577.1346	577.1350	0.69	451.1026, 425.0873, 407.0768, 289.0715	3 dpi
18	theasinensin A	5.71	polymerized catechin derivatives	[M−2H]^2−^	C_44_H_34_O_22_	913.1463	913.1446	−1.86	743.1231, 591.1140, 169.0142, 125.0243	1 dpi
19	unknown	5.72	unknown	[M−2H]^2−^	C_26_H_43_O_36_	930.1456	930.1450	−0.65	ND	1, 3 dpi
20	(E)GC-(E)CG dimer	5.86	polymerized catechin derivatives	[M−H]^−^	C_37_H_30_O_17_	745.1405	745.1399	−0.81	593.1121, 423.0715, 169.0145, 125.0249	1, 3 dpi
21	theasinensin D	5.90	polymerized catechin derivatives	[M−H]^−^	C_44_H_34_O_22_	913.1463	913.1435	−3.07	423.0709, 285.0396, 169.0140, 125.0241	3, 6 dpi
22	procyanidin trimer	6.00	polymerized catechin derivatives	[M−H]^−^	C_45_H_38_O_18_	865.1980	865.1980	0.00	739.1656, 713.1496, 695.1396, 413.0866, 245.0472	3 dpi
23	carthamidin diglucoside	6.03	flavonol glycosides	[M−H]^−^	C_27_H_32_O_16_	611.1612	611.1628	2.62	491.1191, 449.1241, 397.0771	1, 3 dpi
24	isovitexin glucoside	6.03	flavone glycosides	[M+H]^+^	C_27_H_30_O_15_	595.1663	595.1656	−1.18	433.1122, 313.0711	1 dpi
25	(E)GC+(E)C+(E)C isomer 3	6.10	polymerized catechin derivatives	[M−H]^−^	C_45_H_38_O_19_	881.1929	881.1922	−0.79	423.0712, 305.0658, 287.0557, 125.0240	3 dpi
26	unknown	6.10	unknown	[M−H]^−^	C_38_H_32_O_19_	791.1460	791.1453	−0.88	ND	1 dpi
27	diglucopyranosyl trihydroxyflavanone	6.12	flavanone glycosides	[M−H]^−^	C_27_H_32_O_15_	595.1663	595.1667	0.67	475.1249, 433.1353, 313.0934	1, 3 dpi
28	epicatechin *	6.23	flavanols	[M−H]^−^	C_15_H_14_O_6_	289.0712	289.0722	3.46	245.0820, 203.0710, 123.0451	1 dpi
29	unknown	6.30	unknown	[M−H]^−^	C_19_H_30_O_8_	385.1862	385.1854	−2.08	153.0916	1 dpi
30	epigallocatechin gallate *	6.35	flavanols	[M−H]^−^	C_22_H_18_O_11_	457.0771	457.0788	3.72	305.0669, 169.0152, 125.0248	1, 3, 6 dpi
31	theacitrin C	6.36	polymerized catechin derivatives	[M−H]^−^	C_44_H_32_O_22_	911.1307	911.1317	1.10	453.0459, 231.0278	1, 3, 6 dpi
32	unknown	6.37	unknown	[M−H]^−^	C_13_H_18_O_4_	227.0344	227.0347	1.32	ND	1, 3 dpi
33	unknown	6.38	unknown	[M−H]^−^	C_19_H_32_O_8_	387.2019	387.2006	−3.36	ND	1, 3 dpi
34	unknown	6.38	unknown	[M−H]^−^	C_38_H_36_O_19_	795.1773	795.1773	0.00	ND	1 dpi
35	unknown	6.50	unknown	[M−H]^−^	C_38_H_32_O_19_	791.1460	791.1457	−0.38	ND	1 dpi
36	(E)CG+(E)C isomer 1	6.70	polymerized catechin derivatives	[M−H]^−^	C_37_H_30_O_16_	729.1456	729.1457	0.14	577.1154, 407.0759, 289.0709, 125.0242	3 dpi
37	unknown	6.72	unknown	[M−H]^−^	C_38_H_32_O_19_	791.1460	791.1459	−0.13	ND	1 dpi
38	theasinensin F	6.75	polymerized catechin derivatives	[M−2H]^2−^	C_44_H_34_O_21_	897.1514	897.1505	−1.00	575.1186	3 dpi
39	(E)CG+(E)C isomer 2	6.80	polymerized catechin derivatives	[M−H]^−^	C_37_H_30_O_16_	729.1456	729.1446	−1.37	577.1193, 407.0758, 289.0710, 125.0249	1, 3 dpi
40	myricetin 3-neohesperidoside	6.96	flavonol glycosides	[M−H]^−^	C_27_H_30_O_17_	625.1404	625.1405	0.16	316.0224	1, 3 dpi
41	myricetin 3’-glucoside	7.15	flavonol glycosides	[M−H]^−^	C_21_H_20_O_13_	479.0826	479.0829	0.63	316.0225	1 dpi
42	unknown	7.35	unknown	[M−H]^−^	C_39_H_50_O_25_	917.2563	917.2569	0.65	ND	1 dpi
43	quercetin 3-*O*-glucosyl rutinoside	7.39	flavonol glycosides	[M−H]^−^	C_33_H_40_O_21_	771.1984	771.1988	0.52	609.1451, 463.0621, 301.0350, 300.0278	1 dpi
44	epigallocatechin 3-(3-*O*-methylgallate) *	7.43	flavanols	[M−H]^−^	C_23_H_20_O_11_	471.0927	471.0932	1.06	287.0556, 269.0453	1, 6 dpi
45	digalloylprocyanidin dimer	7.53	polymerized catechin derivatives	[M−H]^−^	C_44_H_34_O_20_	881.1565	881.1568	0.34	729.1407, 711.1342, 169.0141	3 dpi
46	rutin *	7.73	flavonol glycosides	[M−H]^−^	C_27_H_30_O_16_	609.1456	609.1457	0.16	533.1294, 300.0277, 271.0251, 255.0299, 243.0298	1, 3, 6 dpi
47	kaempferol 3-*O*-galactosyl rutinoside	7.75	flavonol glycosides	[M−H]^−^	C_33_H_40_O_20_	755.2035	755.2030	−0.66	533.1287, 285.0403	1 dpi
48	epicatechin 3-*O*-gallate *	7.85	flavanols	[M−H]^−^	C_22_H_18_O_10_	441.0822	441.0833	2.49	331.0458, 289.0720, 169.0146, 125.0247	3 dpi
49	tricetin	7.92	flavones	[M+H]^+^	C_15_H_10_O_7_	303.0505	303.0504	−0.33	285.0421	1, 6 dpi
50	quercetin 3-*O*-galactoside	7.93	flavonol glycosides	[M−H]^−^	C_21_H_20_O_12_	463.0877	463.0881	0.86	300.0276	1 dpi
51	kaempferol 3-*O*-glucosyl rutinoside	8.02	flavonol glycosides	[M−H]^−^	C_33_H_40_O_20_	755.2035	755.2036	0.13	285.0404	1 dpi
52	quercetin 3-*O*-glucoside *	8.04	flavonol glycosides	[M−H]^−^	C_21_H_20_O_12_	463.0877	463.0872	−1.08	301.0344, 300.0272	1, 3 dpi
53	capilliposide I isomer	8.17	flavonol glycosides	[M−H]^−^	C_48_H_56_O_27_	1063.2931	1063.2919	−1.13	917.2332, 755.1826, 609.1454, 377.0873, 301.0343, 300.0270	3 dpi
54	unknown	8.39	unknown	[M−H]^−^	C_24_H_42_O_11_	505.2649	505.2645	−0.79	551.2706	1, 3 dpi
55	kaempferol 3-*O*-rutinoside *	8.46	flavonol glycosides	[M−H]^−^	C_27_H_30_O_15_	593.1506	593.1504	−0.34	1187.3071, 285.0398	1, 3 dpi
56	unknown	8.58	unknown	[M−H]^−^	C_20_H_36_O_9_	419.2281	419.2269	−2.86	355.1054	1 dpi
57	epiafzelechin 3-gallate	8.94	flavanols	[M−H]^−^	C_22_H_18_O_9_	425.0873	425.0877	0.94	273.0763, 255.0652	6 dpi
58	sarmenoside II isomer 1	10.31	flavonol glycosides	[M−H]^−^	C_42_H_46_O_22_	901.2402	901.2404	0.22	755.1822, 609.1461, 301.0351, 300.0274	1, 3 dpi
59	theaflavin *	10.59	polymerized catechin derivatives	[M−H]^−^	C_29_H_24_O_12_	563.1190	563.1193	0.53	425.0883, 269.0456, 137.0246	1 dpi
60	sarmenoside II isomer 2	10.91	flavonol glycosides	[M−H]^−^	C_42_H_46_O_22_	901.2402	901.2402	0.00	755.1823, 609.1449, 301.0346, 300.0270	1, 3 dpi
61	theaflavin 3-gallate *	10.97	polymerized catechin derivatives	[M−H]^−^	C_36_H_28_O_16_	715.1299	715.1302	0.42	563.1178, 423.2219, 125.0244	1, 3 dpi
62	theaflavin 3,3’-digallate *	11.17	polymerized catechin derivatives	[M−H]^−^	C_43_H_32_O_20_	867.1409	867.1413	0.46	697.1193, 389.0657, 178.8423	1, 3, 6 dpi
63	theaflavin-3’-gallate *	11.21	polymerized catechin derivatives	[M−H]^−^	C_36_H_28_O_16_	715.1299	715.1287	−1.68	563.1173, 125.0238	1, 3 dpi
64	unknown	13.78	unknown	[M−H]^−^	C_33_H_54_O_15_	689.3384	689.3376	−1.20	671.3262, 653.3162	0 dpi

* Confirmed by comparison with authentic standards. ND, not determined.

## Data Availability

The data presented in this study are available on request from the corresponding author.

## Supplementary Materials

The following supporting information can be downloaded at: https://www.mdpi.com/article/10.3390/metabo13111122/s1, Figure S1: representative total ion chromatograms of mock-inoculated and fungal-inoculated tea leaves at 1 dpi in the negative ionization mode; Figure S2: OPLS-DA analysis of metabolomics datasets; Figure S3: qRT-PCR validation of RNA-seq DEGs; Figure S4: gene ontology (GO) enrichment analysis of differentially expressed genes in gray blight-infected tea plants at 1, 3 and 6 dpi; Table S1: list of primers for qRT-PCR; Table S2: the quality of RNA-seq data; Table S3: gene detected in all samples; Table S4: KEGG enrichment analysis.
