# Supplementary material for: Metabolic Rewiring in Tea Plants in Response to Gray Blight Disease Unveiled by Multi-Omics Analysis
Source: Metabolites. 2023 Nov 1;13(11):1122. doi: 10.3390/metabo13111122 (PMC10672999; doi:10.3390/metabo13111122)
Supplement: Supplementary file 1 [file metabolites-13-01122-s001.zip › Supplemental materials.pdf]

Supplementary Materials

Metabolic Rewiring in tea plants in Response to Gray Blight Disease Unveiled by Multi-Omics Analysis

Shiqin Zheng [1,2,3], Zhenghua Du [2], Xiaxia Wang [2], Chao Zheng [2], Zonghua Wang [3,4,*] and Xiaomin Yu[2,*]

[1]Tea Research Institute, Fujian Academy of Agricultural Sciences, Fuzhou 350013, China

[2]Center for Plant Metabolomics, Haixia Institute of Science and Technology, Fujian Agriculture and Forestry University, Fuzhou 350002, China

[3]State Key Laboratory of Ecological Pest Control for Fujian and Taiwan Crops, College of Plant Protection, Fujian Agriculture and Forestry University, Fuzhou 350002, China

[4] Fuzhou Institute of Oceanography, Minjiang University, Fuzhou 350108, China

* **Correspondence**: wangzh@fafu.edu.cn (Z.W.); xmyu0616@fafu.edu.cn (X.Y.)

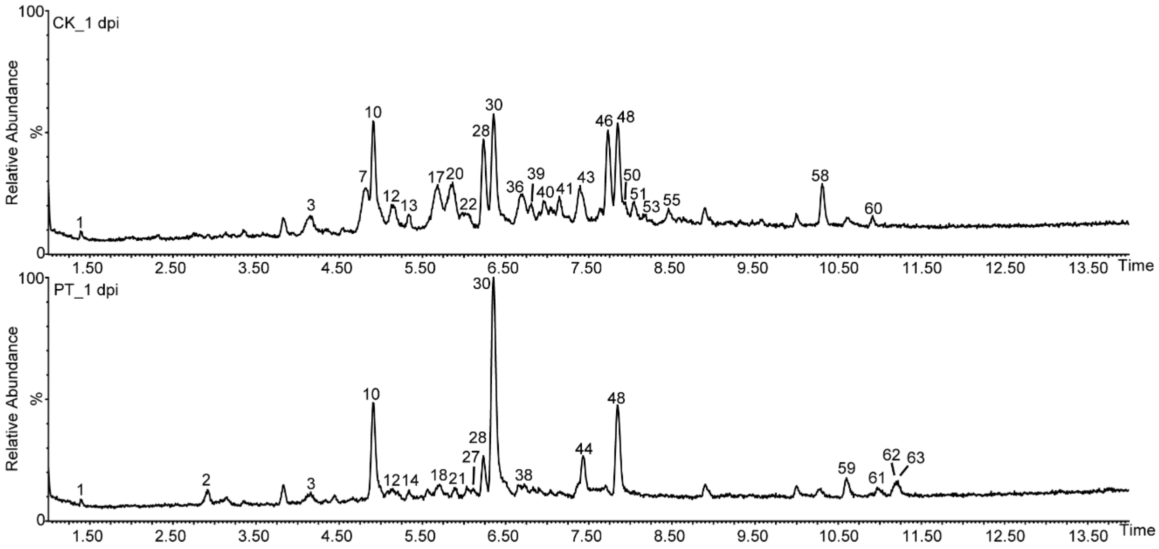

Figure S1. Representative total ion chromatograms of mock-inoculated and fungal-inoculated tea leaves at 1 dpi in the negative ionization mode. PT, tea leaves inoculated with *P. theae*. CK, tea leaves inoculated with the mock control. Peak numbering is the same as Table 1.

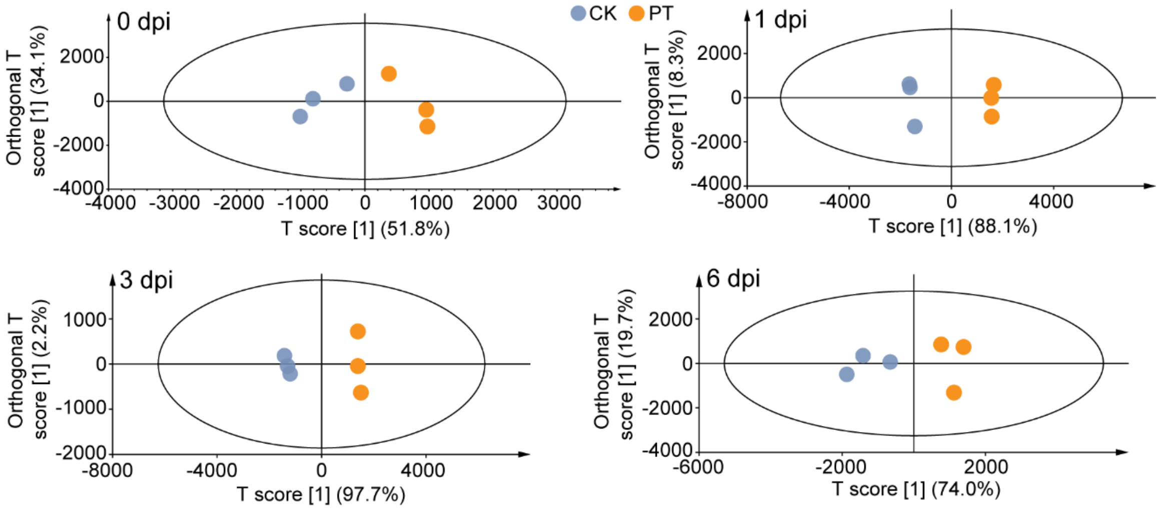

Figure S2. OPLS-DA analysis of the metabolomics datasets. PT, tea leaves inoculated with *P. theae*. CK, tea leaves inoculated with the mock control.

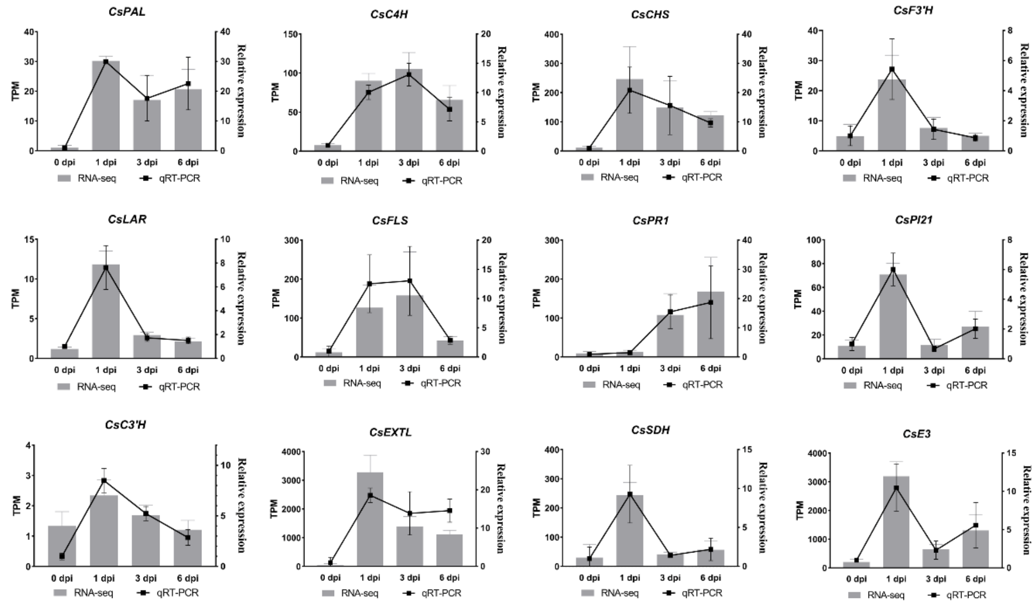

Figure S3. qRT-PCR validation of RNA-seq DEGs. PAL, phenylalanine ammonia-lyase; C4H, cinnamate-4-hydroxylase; CHS, chalcone synthase; F3′H, flavonoid 3′-hydroxylase; LAR, leucocyanidin reductase; FLS, flavonol synthase; PR1, pathogenesis-related protein 1; PI21, PYRICULARIA ORYZAE RESISTANCE 21 ; C3′H, 5-*O*-(4-coumaroyl)-D-quinate 3′-monooxygenase; EXTL, extension-like; SDH, shikimate dehydrogenase; E3, E3 ubiquitin ligase. The left Y-axis represents the expression data (gray bars) and the right Y-axis represents the relative expression values obtained through RT-qPCR analysis (black lines). *GADPH* gene (accession no. KA295375.1) was used as the internal control. The expression of genes in 0 dpi is set to 1.0. All values are mean ± SD (n = 3).

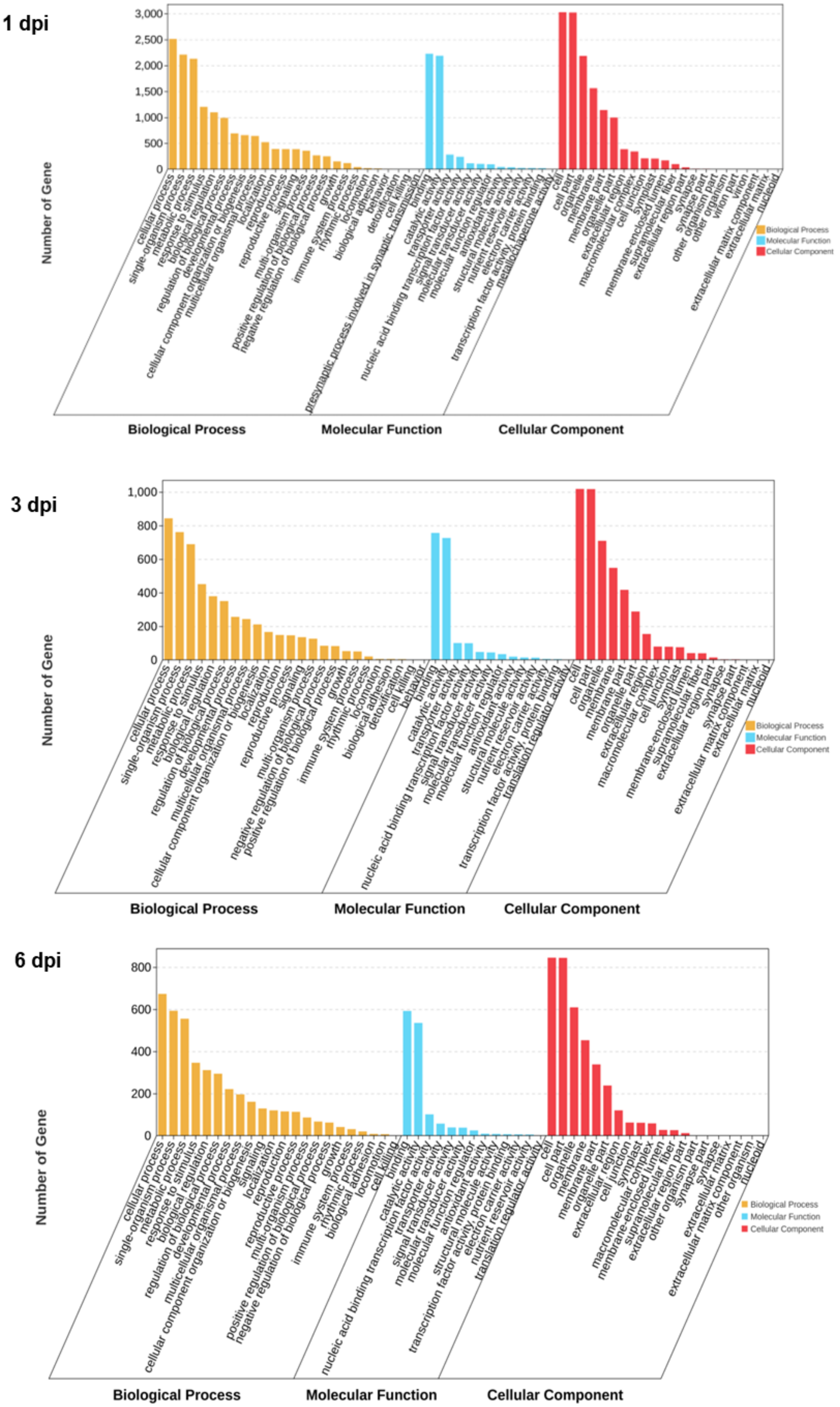

Figure S4. Gene ontology (GO) enrichment analysis of differentially expressed genes in gray blight-infected tea plants at 1, 3 and 6 dpi.

Table S1. List of primers for qRT-PCR.

Target Gene[a]	Forward primer (5′-3′)	Reverse primer (5′-3′)
CsPAL	TCCAATTCCTTGCCAATCCTGTAA	ATCAACTGCCTCGGCTGTCTTTCT
CsC4H	TGCAAAGCTCTCTGGCTATG	CCTGAACTCTTCTGGGTTCTTC
CsCHS	GTGCTATCGATGGGCATCTT	AGGGTTGGAATGCCTCATTTA
CsF3'H	GTTTCGACCAGAACGATTCCTACC	ATAACTGGACCATACGCAACCCTA
CsLAR	ACACTCCCTAGAGTAACCATCA	GATGAAGATGTCGTGGGTGAA
CsFLS	TGGAAAGACGGTAATTGGGTAG	ACTCCTGTGAAGAACACTCTTG
CsPR1	TCCTATGCCCAAAACTCACAACAAG	ACCCATCCTCGAACTAGCATACCT
CsPI21	CTACAGGAATCCAAAGAGTCCAA	CATAGCATTGCCCACAACATATC
CsC3'H	CGGCAACCTCTACGACATAAA	CAACTCTGAGCTCGAAACTATCA
CsEXTL	TGTCTCTGAGCTTGCCATTAG	GAGGTGGTGGTGATTTGTAGT
CsSDH	GTATTGCTCCCATCTCCGCTCACTG	ATGACAACCCTCGCTCCTCTGCTT
CsE3	GATGCTGCTACTCGGTTCTT	GGCATGACGGTTACATCATTTC

[a]PAL, phenylalanine ammonia-lyase; C4H, cinnamate-4-hydroxylase; CHS, chalcone synthase; F3′H, flavonoid 3′-hydroxylase; LAR, leucocyanidin reductase; FLS, flavonol synthase; PR1, pathogenesis-related protein 1; PI21, PYRICULARIA ORYZAE RESISTANCE 21 ; C3′H, 5-*O*-(4-coumaroyl)-D-quinate 3′-monooxygenase; EXTL, extension-like; SDH, shikimate dehydrogenase; E3, E3 ubiquitin ligase.

Table S2. The quality of RNA-seq data.

Sample	Clean reads	Q30	GC%	Mapped rate (%)
PT1_0dpi	25805408	93.21	44	87.50
PT2_0dpi	26011094	92.30	45	88.18
PT3_0dpi	36586286	95.14	45	89.61
PT1_1dpi	25979638	89.29	43	86.66
PT2_1dpi	26575605	93.38	43	86.90
PT3_1dpi	26345386	91.98	43	86.09
PT1_3dpi	28349686	90.43	43	86.98
PT2_3dpi	26270282	92.31	43	86.00
PT3_3dpi	27623253	93.89	43	87.12
PT1_6dpi	28009516	91.86	43	86.84
PT2_6dpi	26367665	89.98	43	87.02
PT3_6dpi	26717807	91.03	44	87.32
